# Identification of functional metabolic biomarkers from lung cancer patient serum using PEP technology

**DOI:** 10.1186/s40364-016-0065-4

**Published:** 2016-06-01

**Authors:** Zhenyu Sun, Xiaofeng Chen, Gan. Wang, Liang Li, Guofeng Fu, Matthew Kuruc, Xing Wang

**Affiliations:** The Third Hospital Affiliated to Nantong University School of Medicine, Wuxi, China; Shanghai Huashan Hospital, Fudan University School of Medicine, Shanghai, China; Institute of Environmental Health Sciences, Wayne State University, 259 Mack Avenue, Detroit, MI 48201 USA; Zibo Central Hospital, Zibo, China; Array Bridge Inc, St. Louis, USA; Biotech Support Group, LLC, Monmouth Junction, NJ USA

**Keywords:** Biomarkers, Functional proteomics, Cancer metabolism, Two-dimensional Gel Electrophoresis (2-D Gel), Protein Elution Plates (PEP), Lung cancer, Enzyme profile

## Abstract

**Background:**

Reprogrammed metabolism is a new hallmark of cancer. In many types of cancer, most of the genes in the glycolytic pathway are overexpressed, reflecting an essential shift of metabolism during cancer development. The reprogrammed metabolism contributes to cancer development in multiple ways, from supplying the elevated energy requirement to creating a microenvironment suitable for tumor growth and suppressing the human immune surveillance system.

**Method:**

In this study, a functional proteomics top-down approach was used to systematically monitor metabolic enzyme activities in resolved serum proteins produced by a modified 2-D gel separation and subsequent Protein Elution Plate, a method collectively called PEP.

**Results:**

We found that the enrichment of low abundance proteins with a bead based product called AlbuVoid™^,^ is important to increase the number of observable features and to increase the level of signal achievable from the assay used. From our methods, significant metabolic enzyme activities were detected in both normal and lung cancer patient sera in many fractions after the elution of the 2-D gel separated proteins to the Protein Elution Plate (PEP). Eighteen fractions with the most dramatic metabolic enzyme activity difference between the normal and lung cancer patient sera were submitted for mass spectrometry protein identification. Proteins from the glycolytic metabolic pathway, such as GAPDH along with other proteins not previously annotated to the glycolytic pathway were identified. Further verification with commercially purified GAPDH showed that the addition of purified GAPDH to the metabolic enzyme assay system employed enhanced the enzyme activity, demonstrating that proteins identified from the PEP technology and mass spectrometry could be further verified with biological assay.

**Conclusion:**

This study identified several potential functional enzyme biomarkers from lung cancer patient serum, it provides an alternative and complementary approach to sequence annotation for the discovery of biomarkers in human diseases.

## Background

Lung cancer is one of the most common malignancies and the leading cause in cancer-related fatality. In the U.S. alone, more than 210,000 new lung cancer cases are diagnosed with more than 170,000 deaths resulting from this disease each year. Lung cancer is the 5^th^ leading cause of death worldwide, there were 1.5 million lung cancer deaths in 2010, an increase of 48 % in the past 20 years [32]. Usually symptoms of lung cancer do not appear until the disease is already in an advanced, non-curable stage. Most patients present with advanced disease and 5-year survival rates are poor, ranging from less than 10 % in China to 13–16 % in Europe and the US [32]. Even when symptoms of lung cancer do appear, many people may mistake them for other problems, such as an infection or long-term effects from smoking, delaying the disease diagnosis. Current practice on the diagnosis of common cancers relies heavily on imaging technologies such as CT scans for lung cancer, mammograms for breast cancer and pelvic ultrasounds for ovarian cancer. While advances in imaging technology have allowed more sensitive detection of small lesions, these advances have also led to an increase in false positive findings and invasive procedures to make a definitive diagnosis. For example in lung cancer; the CT scanners have led to the detection of large numbers of small pulmonary nodules. The frequency of detecting noncalcified nodules on a single CT varies from 5 to 60 % in a lung cancer screening population [12]. Given the high probability of false positive findings associated with CT screening, there is a substantial need for additional noninvasive modalities to discriminate between benign and malignant nodules. There are similar challenges in imaging based screening for other malignancies and a subsequent need for complementary diagnostic tests.

Blood based biomarkers have potential in cancer screening and their role could extend further from general population risk assessment to treatment response evaluation and recurrence monitoring. The rich content of diverse cellular and molecular elements in blood, which provide information about the health status of an individual, make it an ideal compartment to develop noninvasive diagnostics for cancer [42]. However, despite a large literature collection related to biomarkers for common cancers, blood based diagnostic tests that inform about the presence of cancer at an early stage and predict treatment response have been difficult to develop [24, 47]. Protein markers currently in clinical use, which include CA125 (cancer antigen 125) for ovarian cancer, CA199 (carbohydrate antigen 199) for pancreatic cancer, CEA (carcino embryonic antigen) for colon cancer and PSA (prostate specific antigen) for prostate cancer, have limitations with respect to their use for screening owing to low sensitivity and specificity in early stages and inability to distinguish aggressive from indolent tumors [11]. Other common cancers, notably breast and lung cancer, lack established biomarkers with demonstrated clinical utility in a screening setting. Thus, there is a need for biomarkers with the required sensitivity and specificity for the detection of frequently occurring cancer types [24].

Over the past decade, system biology especially proteomics has been used for the discovery of potential biomarkers from human fluids including serum [1, 15, 20, 22, 40, 42, 46]. So far, most efforts in proteomics seek to identify and sequence annotate the proteome by mass spectrometry analyses of peptides derived through proteolytic processing of the parent proteome [25, 32, 33, 35]. In such manner, thousands of proteins have been identified from human serum (www.serumproteome.org). However no validated protein biomarker currently exists for use in routine clinical practice for lung cancer early detection, prognosis and the prediction of treatment response. Proteomic profiling could potentially provide such markers.

One of the challenges from mass spectrometry-based proteomics is to overcome the analytical bias towards the most abundant serum proteins, and the complexities of mining the data to a manageable number of biomarker proteins that can be analyzed in more depth. Currently in proteomics research, little attention has been paid to systematic functional annotation, yet functional annotation is crucial as many proteins have variants and each functional variant forms may contribute to its own unique functional activity. It is generally recognized that sequence annotation alone cannot capture this vital information, so new strategies are necessary. So reconciling protein identifications to actual enzyme activities or functions has been subject to limitations in proteome separation and assay technologies. To overcome these inefficiencies in functional annotation, a top-down approach, starting with function, and ending with sequence and structural annotation and functional validation was developed. The PEP technology uses a modified Two-dimensional Gel Electrophoresis to separate the proteome, without substantially compromising function [19]. The isolated proteins are then electro-eluted from the PEP plate and further refolded, and enzyme activities are measured systematically from hundreds to thousands of fractions depending on the complexity of the proteome. This method thus provides a new functional dimension to explore the human serum proteome.

Human serum contains thousands of proteins with abundances spanning eight logs [36]. In the past decade thousands of proteins have been identified from the human serum mainly by mass spectrometry technology and many of them are enzymes supporting catalytic function within cells (www.serumproteome.org). However, for the majority of proteomic applications, antibody-based detection, 2-D gel electrophoresis followed by protein staining, and quantitative label and label-free LC-MS, are used to measure protein abundances and carry out the comparison. Differential gel electrophoresis (DIGE) is a common method to pair disease sample and control with two different fluorescence dye staining to study relative protein up or down regulation, and this approach has been applied to the identification of potential drug targets or biomarkers [1, 42]. However, the functional differences between the disease and normal proteomes can’t be analyzed with approaches solely reliant on protein abundances, as conformational variations produce functions, not strictly proportional to the abundances of the gene derivative polypeptide products. As a result, there is a distinct advantage in characterizing the functions of a proteome.

Many functional assays are very sensitive especially those based on fluorescence detection, being able to measure enzyme activities at picogram level [14, 23, 41, 43, 45]. This is significantly more sensitive than the most sensitive detection methods from electrophoretic gels with silver staining or fluorescence staining, or the general mass spectrometry methods. Secondly, it is known that many proteins have post-translational modifications (PTMs) and splice variants [2], these different forms of the same gene product may have different functions/enzyme activities and play very different roles in biology. However the important information on the impact of PTM and protein splicing is lost in the antibody, LC-MS or gel-based analysis because these platforms cannot directly measure the functional features of the proteome. New methods to monitor and compare functional proteomes are therefore desirable.

It is hypothesized that the levels and distributions of certain enzyme functions in serum could produce proteomic features and collective profiles which reflect physiological changes of an individual and can serve as possible biomarkers or diagnostic parameters. To achieve such features, a modified 2-D gel electrophoresis (2-DE) system can be used; 2-DE being a powerful tool to separate proteomes based on two orthogonal parameters, isoelectric point (pI) and molecular weight respectively. Thousands of protein spots can be detected from a large format 2-D gel. However, the typical 2-D gel electrophoresis includes the use of reducing reagent to disrupt the protein disulfide bonds and the use of high concentration SDS to denature and negatively charge the proteins for the second dimension separation. In the current PEP technology, reducing reagent is not used, keeping the disulfide bonds intact. Furthermore, a much reduced SDS concentration (0.1 %) was used to charge the proteins before the second dimension, this is a 20-fold reduction over the typical SDS treatment. This modified condition allows the proteins to get negatively charged but the condition is not strong enough to destroy the protein tertiary structure. This, in combination with protein refolding and protein protectants in the PEP system, allows the efficient recovery of enzyme activities from the serum proteome after 2-D gel electrophoresis and protein elution [19]. Our initial studies using colon and breast cancer patient serum and normal serum provided strong evidence for a clear disease signature when the enzyme activities were compared (data not shown). Since most of the functional proteins or enzymes exist at relatively low level in the human serum and there is a limited loading capacity on the 2-DE gel, it is important to enrich the low abundance proteins before 2-DE and PEP analysis. AlbuVoid™ (Biotech Support Group, Monmouth Junction NJ) has been shown to effectively enrich low abundance serum proteins while depleting the Albumin. It was used previously for pre-treatment of human serum in the PEP technology, whereby more functional features were observed with AlbuVoid™ than without (data not shown). Consequently, we adopted AlbuVoid™ in our workflows in this investigation.

The selection of the activity to be monitored is an important choice in any functional proteomic investigation. Previous studies have demonstrated that general redox enzymes could be detected from beef liver extract or mouse cochleae tissue, both NADH and NADPH-dependent oxidases can be detected from a large number of fractions after 2-D gel separation and PEP elution [19]. However, the relative enzyme activities were low because instead of specific substrates, general enzyme substrates containing all the 20 amino acids and several sugars were used at relatively low level; the purpose of this approach was to maximize the detection of enzyme species using NADH or NADPH as cofactor. In the current study, a more specific assay for hexokinase activity was measured, with only two substrates (glucose and ATP) introduced at optimized conditions [13]. Hexokinase activity was selected for several reasons. Foremost is that the products produced from Hexokinase activity are the first within the glycolytic pathway, a pathway often implicated in cancer development [13, 21, 37]. Another reason is that a large number of functional proteins within and cross-regulating with the glycolytic pathway could potentially be monitored by a broad spectrum assay as the one employed, which already contains low level of endogenous Hexokinase activity. The introduction of exogenous protein(s) from the PEP samples could potentially supersede any rate-limiting protein function and enhance the hexokinase activity. As such, this assay may also detect the effect of proteins from other pathways that cross-interact with the glycolytic pathway.

## Methods

### Materials

All the chemicals were purchased from Sigma-Aldrich (St. Louis, MO). Isoelectric Focusing (IEF) unit that is capable of running IEF at different length is from Bio-Rad (PROTEAN IEF Cell). Spectrophotometer Plate Reader capable of reading 384-well plates with a wide wavelength selection and fluorescence reading is the SPECTRAMax Plus from Molecular Devices (Sunnydale, CA). Semi-Blot unit for protein transfer such as Bio-Rad’s Trans-Blot SD Semi-Dry Transfer Cell.

### Beef liver protein extract preparation

Frozen beef liver tissue purchased from the local supermarket. 5 g of frozen beef liver tissue was chopped up with a razor blade and homogenized in 2.5 volumes of phosphate-buffered saline (PBS) using a disposable plastic homogenizer. After spinning at 14,000 g for 15 min, the supernatant was analyzed for protein concentration with BCA and frozen at −20 °C in small aliquots for single use.

### AlbuVoid™ treatment for low abundance serum protein enrichment

200 mg of AlbuVoid™ beads were used to process 0.8 ml of human serum (contains about 40 mg total serum protein). 20 serum samples from normal people or lung cancer patient were pooled with equal volume (100 μl each) respectively and separated with AlbuVoid™ according to the manufacturer’s instruction. The patient serum was collected at Wuxi Third People’s Hospital in China after the approval from the Hospital Ethics Committee with reference number of 20140102 AlbuVoid™ will not bind serum albumin, thus this most abundant serum protein is removed in the flow through fraction and the rest of serum proteins are enriched on the AlbuVoid™ column. The enriched low abundance serum proteins were eluted with 0.8 ml elution solution containing 8 M urea, 2 % CHAPS in 25 mM phosphate buffer, pH 8.0. The protein concentration was determined by BCA before 2-D gel electrophoresis.

### Isoelectric Focusing (IEF) and 2-D gel electrophoresis

To prepare for the IEF separation, Bio-Lyte Ampholyte (Bio-Rad #1631112) was added to the elute above for a final concentration of 0.5 %, rehydrate with 0.4 ml sample solution with nonlinear pH 3–10 11 cm IPG strip (Bio-Rad ReadyStrip #1632016) overnight with a total loading of 1 mg protein/gel. The proteins were separated using the following setting: 0–7000 voltage gradient for 4 h, Hold at 7000 voltages overnight until running termination, the IEF was run at room temperature. After IEF, the IPG strips were taken off the running unit, mineral oil from the IPG strip was absorbed with a paper towel and the IPG strip was transferred to a 12-lane refold tray (Bio-Rad #1654025). 4 ml refolding solution was added to each lane with the IPG strip and incubated for 10 min., this step will allow the urea to diffuse out of the IPG strip and also the refolding of the protein in the IPG strip, this was followed by incubation with electrophoresis transfer buffer (Tris-glycine with 0.1 % SDS), this step will allow the further diffusion of urea from the IPG strip and most importantly the binding of SDS to the protein so that all the proteins were negatively charged.

For protein refolding, a proprietary protein refolding solution was used; the solution contains multiple metal elements to replace the possible loss of metal ions as enzyme cofactors. A redox system to mimic the cell cytoplasm was used to assist the protein refolding process. After protein refolding, the IPG strip was laid down to a precast 2-D gel (Bio-Rad 10 %-20 % Criterion Gel #3450107) with the acidic end of the IPG on the left side of the 2-D gel when facing the gel apparatus. The gel was operated at 80 volts for 15 min. followed by running at 120 voltages until the dye front of the gel is 0.5 cm from the bottom edge of the gel.

### Electroelution and protein recovery from the PEP plate

After second dimension gel electrophoresis, the gel was taken out from the cassette, and laid on top of the PEP plate which was filled with elution solution. The proteins were transferred from the gel to the PEP plate for 60 min. at 20 volts using a Semi-Blot apparatus from Bio-Rad (#1703940.). After protein transfer, the gel was carefully lifted from the PEP plate, and a multi-channel pipette transferred the eluted proteins from the PEP plate to a master plate, containing 50 μl PBS in each well. About 40–45 μl of solution could be transferred to the Master Plate for a total volume of 90–95 μl in each well. In this analysis, 25 μl solution was taken from each well in the Master Plate and transferred to an enzyme assay plate to perform the enzyme assay and the remaining solution was used for mass spectrometry protein identification.

### Hexokinase activity assay

Hexokinase activity can be monitored by a cascade reaction as follows:

$$ \mathrm{Substrates}\ \mathrm{added}\ \left\{\mathrm{D}\hbox{-} \mathrm{Glucose}+\mathrm{A}\mathrm{T}\mathrm{P}\right\}\overset{\mathrm{Hexokinase}}{\to}\mathrm{P}\mathrm{roducts}\ \left\{\mathrm{D}\hbox{-} \mathrm{Glucose}\ 6\hbox{-} \mathrm{Phosphate}+\mathrm{A}\mathrm{D}\mathrm{P}\right\} $$

$$ \mathrm{D}\hbox{-} \mathrm{Glucose}\ 6\hbox{-} \mathrm{P}\mathrm{hosphate} + \mathrm{\ss}\hbox{-} \mathrm{NADP}\overset{\mathrm{G}\hbox{-} 6\hbox{-} \mathrm{P}\mathrm{D}\mathrm{H}}{\to }6\hbox{-} \mathrm{P}\mathrm{hospho}\hbox{-} \mathrm{D}\hbox{-} \mathrm{Gluconate} + \mathrm{\ss}\hbox{-} \mathrm{NADP}\mathrm{H} $$

In the final assay solution, glucose was at 216 mM; MgCl2 at 7.8 mM, ATP at 0.74 mM and NADP at 1.1 mM. 25 μl of this enzyme assay solution was mixed with 25 μl of sample from the Master Plate (described above) and the enzyme activity was monitored by the 340 nm absorbance from the reduction of NADP to NADPH. The readings at 0, 1 h., 3 h. and overnight were recorded for both the normal serum and lung cancer patient serum sample. However, in lieu of purified G-6-PDH, 0.25 mg/ml beef liver protein was used as the source of Glucose-6-Phosphate Dehydrogenase (G-6-PDH). The assay thus reports the additive contributions of the endogenous Hexokinase activity present in the beef liver extract, and any exogenous activity from the presence of test sera protein in the PEP plate, which may influence the reduction of NAD or NADP (the reporting signal). In light of the ambiguities that may arise from such a reporting system, the primary goal of this investigation was to generate sufficient signal intensities and activity features which could monitored and compared across samples types within an ‘omics’ context. Therefore, this broader spectrum assay was chosen over a more narrow spectrum substrate to product turnover one, as this broader spectrum assay could potentially detect the activities of downstream glycolytic and other cross-regulating proteins from the test sera.

### Enzyme activity display

Two Microsoft Excel formats were used to display the enzyme activities. One is to use the 3-D column display and the other is the heat map.

### Protein identification by LC-MS/MS

For fractions with significant hexokinase activity and also demonstrated qualitative or quantitative differences between the normal serum and lung cancer patient serum, sample was recovered from the Master Plate and treated with 50 μl 20 % cold TCA (Trichloroacetic Acid) to remove non-protein components and recover the proteins from the fraction. The precipitate was washed twice with cold acetone to remove the TCA and dried in a SpeedVac for 60 min. The dried samples were submitted for Mass Spectrometry protein identification. For MS analysis, the samples were loaded onto a 10 % bis-Tris NeuPage gel as a gel plug. The gel plug was excised and proteins in the gel were reduced, carboxymethylated, digest with trypsin using standard protocols. Peptides were extracted, solubilized in 0.1 % trifluoroacetic acid, 5 % acetonitrile, and analyzed by nanoLC-MS/MS using a RSLC system (Dionex, Sunnyvale CA) interfaced with a Velos-LTQ-Orbitrap (ThermoFisher, San Jose, CA). Samples were loaded onto a self-packed 100 μm × 2 cm trap packed with Magic C18AQ, 5 μm 200 A (Michrom Bioresources Inc, Aubum, CA) and washed with Buffer A (0.2 % formic acid) for 5 min with flowrate of 10 μl/min. The trap was brought in-line with the homemade analytical column (Magic C18AQ, 3 μm 200 A, 75 μm × 50 cm) and peptides fractionated at 300 nL/min with a multi-stepped gradient (4 to 15 % Buffer B (0.16 % formic acid 80 % acetonitrile) in 10 min and 15–35 % B in 55 min, and 30–55 % B in 25 min). Mass spectrometry data was acquired using a data-dependent acquisition procedure with a cyclic series of a full scan acquired in Orbitrap with resolution of 60,000 followed by MSMS scans (acquired in linear ion trap) of 20 most intense ions with a repeat count of two and the dynamic exclusion duration of 60 s.

The LC-MSMS data was searched against the ensemble human database (most updated) using X!Tandem through a local version of the Global Proteome Machine (GPM cyclone, Beavis Informatics Ltd, Winnipeg, Canada) with carbamidoethyl on cysteine as fixed modification and oxidation of methionine and tryptophan as variable modifications using a 10 ppm precursor ion tolerance and a 0.4 Da fragment ion tolerance.

## Results and discussion

For the enzyme activity profiles to produce distinguishable features which can be compared, three necessary components of the method were first validated:

1. Low Abundance Serum Proteins can be enriched with the AlbuVoid™ beads.
2. 2-DE separation can resolve proteins with subsequent transfer to PEP multi-well plates, and
3. The Hexokinase assay can monitor refolded proteins so as to generate a detectable signal over and above background signal from the activity assay.

### Establish low abundance protein enrichment from human serum

**Fig. 1:**
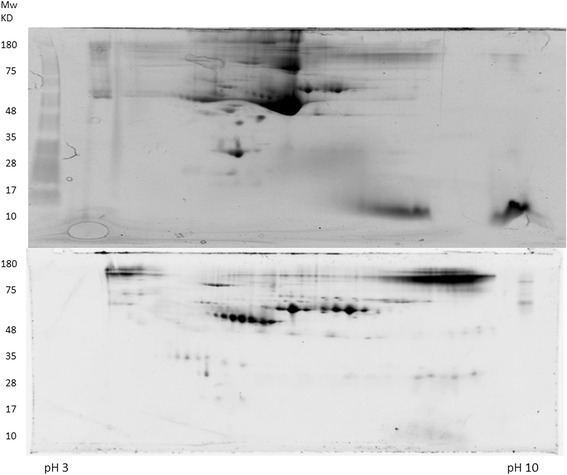
Enrichment of Low Abundance Proteins from Human Serum. 800 μl pooled serum from normal (*N* = 20) and lung cancer patient (*N* = 20) was used for the enrichment of low abundance proteins with AlbuVoid treatment according to the manufacturer’s instruction. For the comparison, 750 μg protein from untreated serum (normal group) and treated serum (normal group) was loaded onto two 11 cm IPG strips for IEF protein separation without reducing reagent. For second dimension separation, SDS-PAGE with 10–20 % gradient gel was used, Sypro Ruby was used for protein staining and detection

In order to achieve the maximum sensitivity of the PEP technology, 800 μl of serum (about 40 mg total protein) was used for the AlbuVoid™ treatment. As can be seen in Fig. 1, the major serum protein- Albumin, was not bound to the AlbuVoid™ beads and more low abundance proteins are detectable. This enrichment process will increase the enzyme assay sensitivity by least 10-fold compared with direct analysis without enrichment.

### Establish 2-DE resolution

**Fig. 2:**
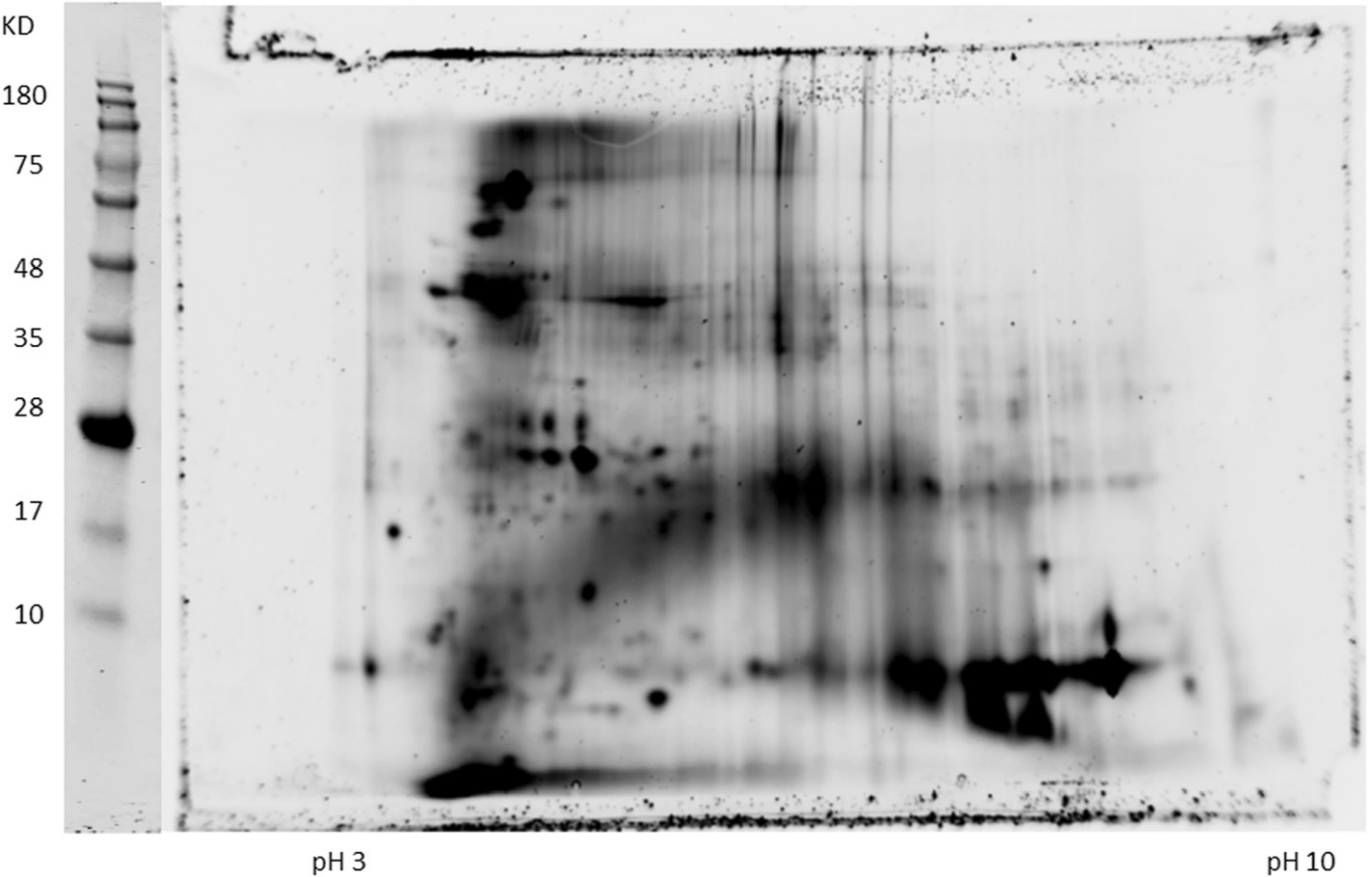
Effective 2-D Gel Separation of Beef Liver Proteins without Reducing Reagent. 400 μg of beef liver protein was loaded on an 11 cm pH 3-10Nonlinear IPG strip, for the second dimension, the proteins were separated in SDS-PAGE with gradient gel of 10–20 %. After fixing, the proteins were detected by Sypro Ruby staining

Before enzyme analysis, a good 2-D gel separation condition needs to be established. For this, we used the beef liver protein extract to establish the optimum conditions. As can be seen in Fig. 2, at a loading of 400 μg/gel, most of the beef liver proteins were well separated under the modified 2-D gel condition (mainly the exclusion of reducing reagents to retain the disulfide bonds in the protein) and a large number of proteins were detected by fluorescence staining. For the 2-D gel separation of the enriched serum proteins, 1 mg/gel was used with acceptable resolution (Fig. 1). An unstained gel was used to elute proteins directly into a PEP plate. After elution of the proteins from the 2-D gel to PEP plate, hexokinase activities were measured from every well in the 384-well microplate.

### Hexokinase activity analysis from 2-D gel-separated serum proteins

**Fig. 3:**
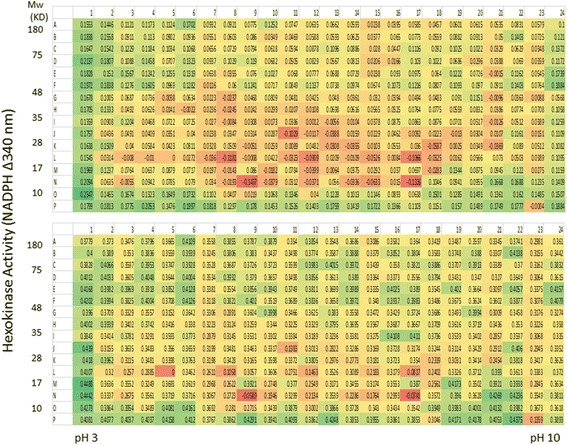
Time-dependent Measurement of Hexokinase Activity from the PEP Platform. After elution of proteins from the 2-D gel into the 384-well PEP plate, the eluted proteins were further transferred into a 384-well Master Plate with 50 μl refolding solution. 25 μl of the solution from the Master Plate was further transferred into a 384-well Enzyme Assay plate, 25 μl of hexokinase assay components were was added to each well, the increase of OD 340 nm by NADP reduction was measured in a spectrophotometer, the OD readings were taken at 0, 60 min. and 120 min., the OD340 nm increase from 60 min. and 120 min. was calculated by subtracting the value from the 0 min. reading. Top panel: ΔOD after 60 min incubation.  Bottom panel: ΔOD after 120 min incubation

It should be pointed out that the refolding process after the isoelectric focusing is critical to regain good enzymatic activity. During IEF, 8 M urea was used to achieve the best 2-D gel separation, however this condition also results in unfolding of the proteins, therefore after 2-D gel electrophoresis, and the proteins need to be refolded. Another consideration during the refolding process is to replenish the metal ions lost during gel electrophoresis. It is known that many enzymes use metal ions as cofactors for their enzymatic activity; therefore it is important that during the refolding process, in addition to the protein refolding system, several metal ions commonly used by enzymes were added to assist the refolding process. In this step, the urea from the IPG strip is diffused out and the proteins would presumably assume the right conformation with the redox system and metal ions provided. Another important aspect of the improved gel electrophoresis is the binding of SDS to the proteins. It is known from previous studies that many proteins are still active in the presence of SDS [5], however higher level of SDS could potentially induce protein inactivation. Therefore a much reduced SDS concentration was used for the sample treatment before the second dimension gel electrophoresis. The rationale is that the SDS treatment will just be enough to negatively charge the protein for an acceptable resolution but not strong enough to induce significant protein denaturation. After electroelution, the eluted proteins from the PEP plate were transferred to a master plate in which protein refolding solution was added for the further refolding of the proteins. As can be seen in Fig. 3, time-dependent hexokinase activities were detected from a large number of fractions.

Since this beef liver extract contains all the enzymes in the glycolytic pathway, any proteins that directly or indirectly impacts the glycolytic pathway or NADP reduction (reporting component of the assay), could be detected in this setting. Therefore the hexokinase activity detected in the current assay is not solely the result of the hexokinase reaction products: Glucose-6-phosphate & ADP, it could be the combined effect of all the enzymatic activities monitored by the increase in spectrophotometric absorbance of NADP reduction. It is interesting to note that as many as 1/3 of 384 PEP fractions from the sera, showed various levels of such activity, suggesting that a large number of proteins from beef liver might be involved in NADP reduction directly or indirectly. Another interesting observation from the hexokinase activity assay on the PEP platform was that the activities observed were much stronger than the NADH or NADPH-dependent enzyme assays before [19], suggesting a more narrow substrate panel is beneficial. The third observation for the hexokinase activity analysis was the wide distribution of fractions with the capability of hexokinase activity enhancement, ranging from molecular size as high as 200 kDa to as low as 5 kDa, and the isoelectric points from 3 to 10, suggesting a wide variety of proteins, not previously annotated to the glycolytic pathway, may nevertheless be servicing this pathway directly or indirectly. With an emerging view that many proteins are multi-functional due to a continuum of conformational transitions, it will be interesting to identify these proteins and understand how they contribute to the glycolytic pathway.

### Comparison of hexokinase activity from normal serum and lung cancer patient serum

**Fig. 4:**
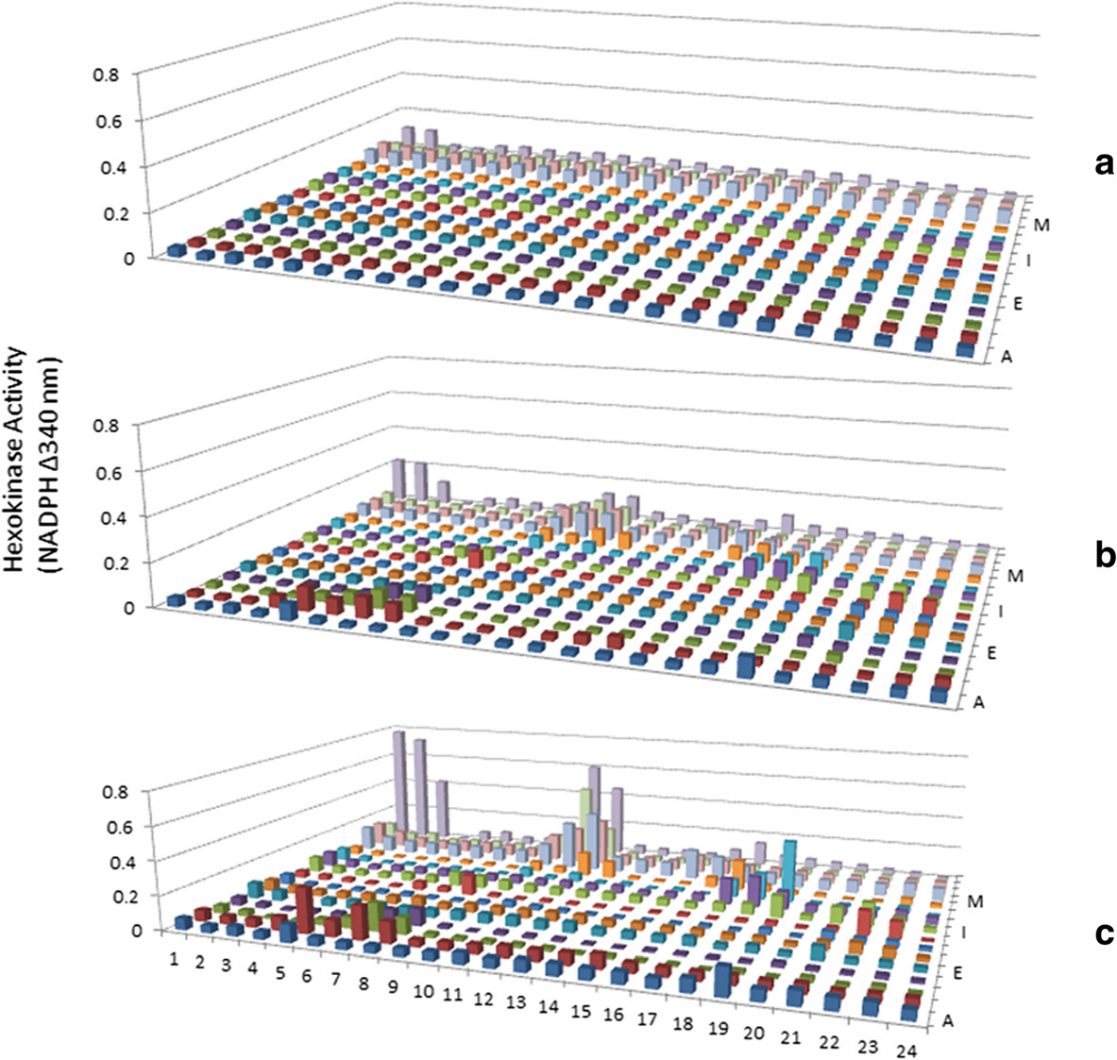
Time-dependent Hexokinase Activity Analysis from AlbuVoid-enriched Normal Serum. 750 μg AlbuVoid-enriched serum proteins from normal group was loaded onto an IPG strip and separated by IEF. After further separation by SDS-PAGE, the proteins were eluted into the PEP plate and hexokinase activities were analyzed from each of the 384 wells. Hexokinase activity was measured by NADP reduction at 340 nm at 0 (*a*), 60 min (*b*). and overnight (*c*) incubation

**Fig. 5:**
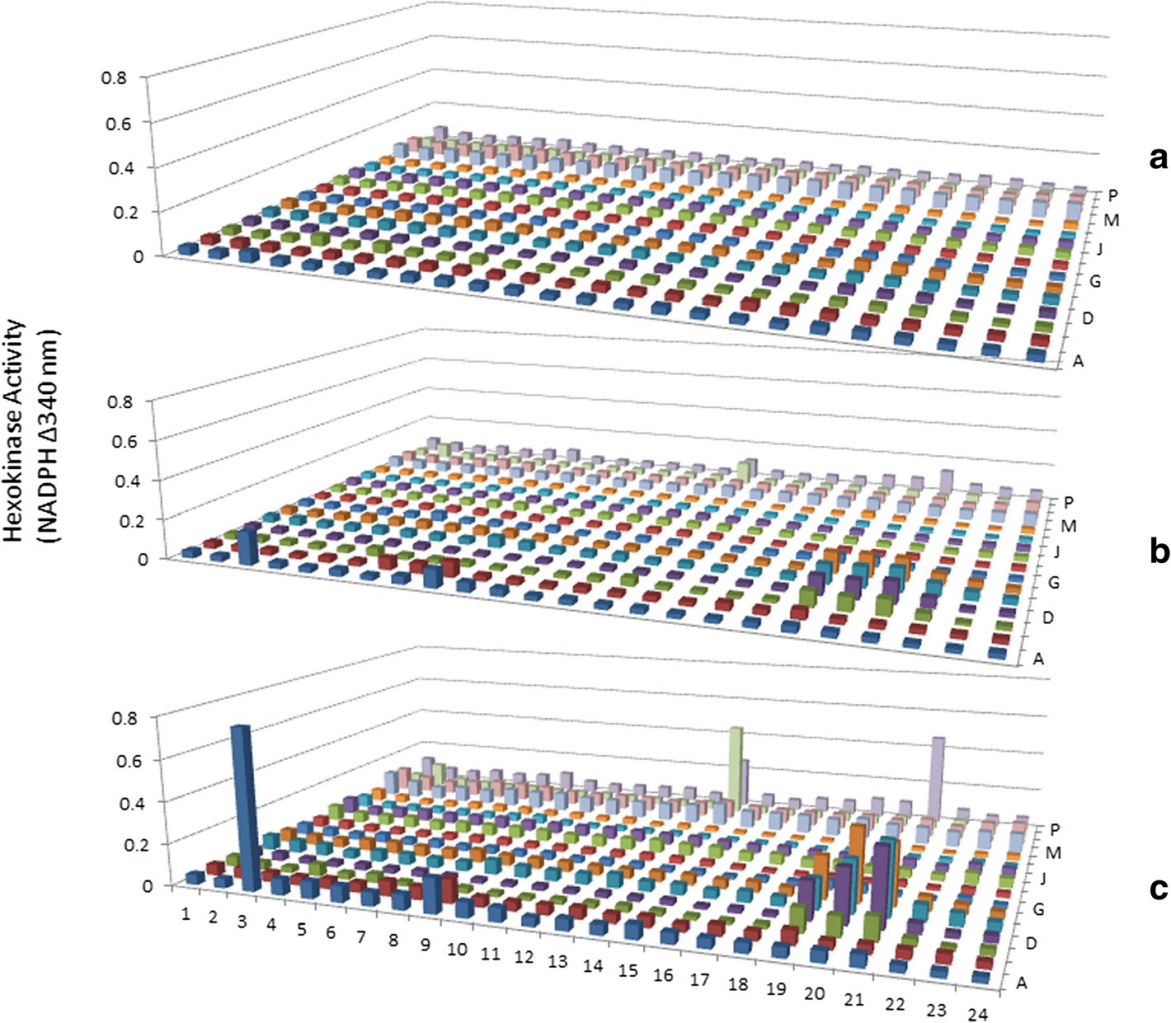
Time-dependent Hexokinase Activity Analysis from AlbuVoid-enriched Lung Cancer Patient Serum. 750 μg AlbuVoid-enriched serum proteins from lung cancer patient group was loaded onto an IPG strip and separated by IEF. After further separation by SDS-PAGE, the proteins were eluted into the PEP plate and hexokinase activities were analyzed from each of the 384 wells. Hexokinase activity was measured by NADP reduction at 340 nm at 0 (*a*), 60 min (*b*). and overnight (*c*) incubation

To demonstrate that hexokinase activity can be monitored from the PEP fractions, the reduction of NADP was measured at different time points. As can be seen in Fig. 4, many fractions from the normal serum have time-dependent hexokinase activities. Similarly, measurements on the serum from lung cancer patients also showed many fractions with hexokinase activities, and more interestingly, there are qualitative and quantitative differences between the normal serum and cancer patient serum (Figs. 4 and 5).

**Fig. 6:**
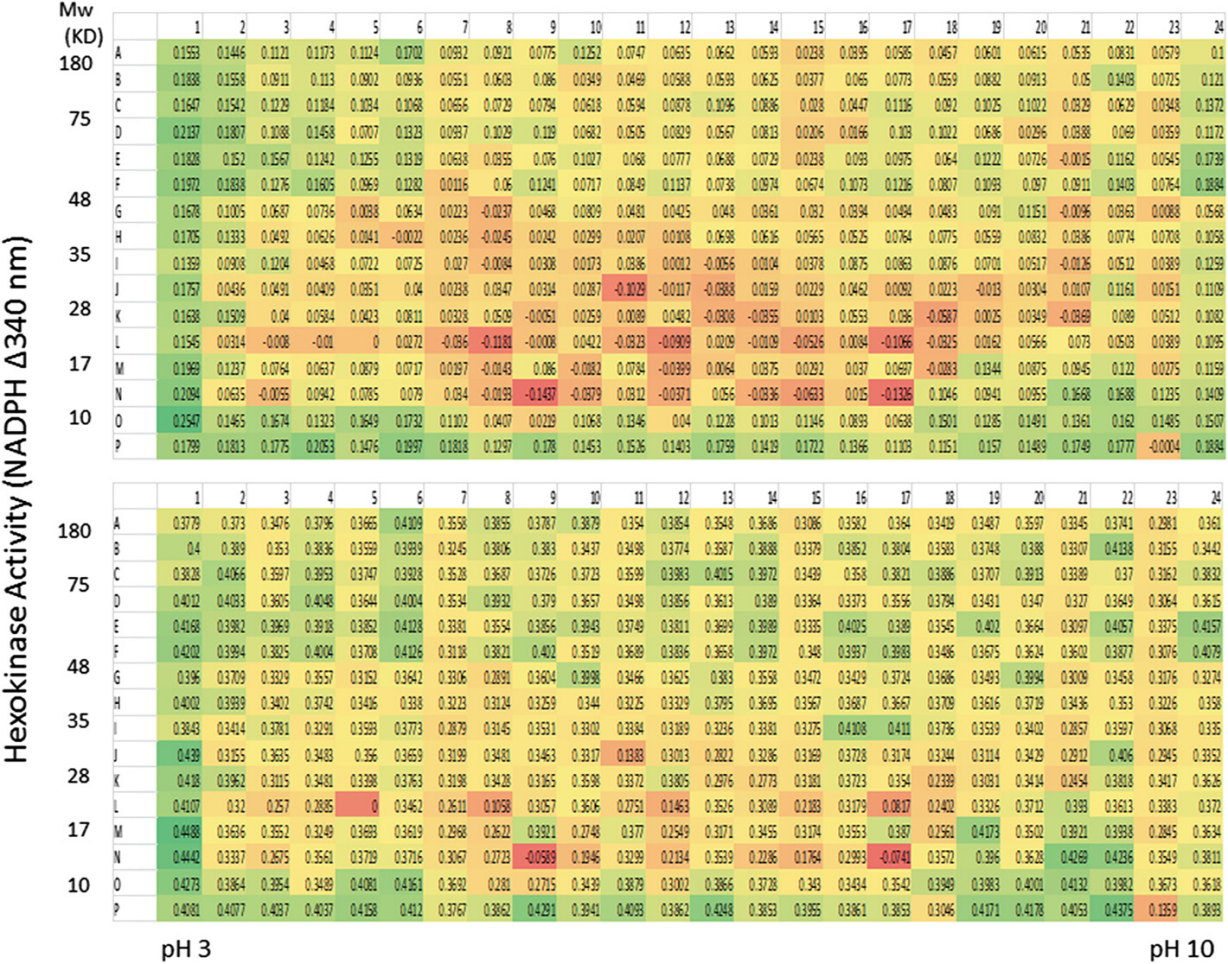
Comparison of Normal and Lung Cancer Patient Serum Hexokinase Activity Pattern. Hexokinase activities from both normal (upper panel) and lung cancer (lower panel) patient serum were displayed in a heat-map for comparison and subsequent process for mass spectrometry identification

For a better comparison of the hexokinase activities between the normal and lung cancer patient sera, a heat map was constructed using Microsoft Excel (Fig. 6). As can be seen, a large number of fractions from both the normal and lung cancer patient serum were found to have hexokinase activity, even though we may not know the precise mechanism for up-regulated activity. Another interesting observation from Fig. 6 was that there were many fractions with significant hexokinase activity differences between the normal and lung cancer group. For example, in the normal serum, K19 and P10 have more than 10-fold hexokinase activity than the corresponding fractions in the lung cancer patient serum. Conversely, from the lung cancer patient serum, fractions A3, D20, E20, F20, O14 and P21 each has about 10-fold more activities than the corresponding fractions from the normal group, and those fractions with enzyme activity showed a time-dependent activity increase. The above mentioned fractions and many other fractions will be good candidates to look for potential biomarkers. Another interesting observation was that the fractions with hexokinase activity were detected across a wide range of molecular size and isoelectric points, suggesting that: 1.) there are many serum proteins that could directly or indirectly impact hexokinase activities within this assay system, and 2.) there could be protein variants that show different hexokinase activity among the fractions. This is the first time that hexokinase activity has been surveyed systematically from a highly resolved proteome.

### Mass spectrometry identification of serum proteins with glycolytic enzyme activity

For the identification of proteins with demonstrated time-dependent hexokinase activity from the enzyme assay plate, the corresponding fractions from the master plate (see Materials and Methods for details) were collected and precipitated by TCA and submitted for mass spectrometry analysis. It is interesting to note that the majority of proteins identified with significant sequence coverage have not previously been annotated to the glycolytic pathway; a potential subject for future investigation. As was shown in Table 1, three known enzymes from the glycolytic pathway, GAPDH, PKM and Enolase, were identified from the MS analysis.

**Table 1:** Proteins identified by mass spectrometry after separation with PEP technology and Hexokinase Activity Analysis

Sample number	Sample location in PEP plate	Tentative protein ID
Sample-1	N19A	Plasminogen (PLG)
Sample-2	N7B, N8B	Hemopexin (HPX)
Sample-3	N22H, N23H, N22I	Complement Component 3 (C3)
Sample-4	N17J, N18J	Fatty Acid Synthase (FASN)
Sample-5	N18K, N19K	Glyceraldehyde-3-phosphate Dehydrogenase (GAPDH)
Sample-6	N10M, N11M	Fatty Acid Synthase (FASN)
Sample-7	N15M, N16M	Enolase 1 (ENO1)
Sample-8	N1P, N2P, N3P	Glyceralde-3-phosphate Dehydrogenase
Sample-9	N10P, N11P	Alpha-2-glycoprotein 1 (AZGP1)
Sample-10	C3A	Fatty Acid Synthase (FASN)
Sample-11	C9A, C9B	Glyceraldehyde-3-phosphate Dehydrogenase (GAPDH)
Sample-12	C19D, C20D, C21D	Pyruvate Kinase (PKM)
Sample-13	C19E, C20E, C21E	Glyceraldehyde-3-phosphate Dehydrogenase (GAPDH)
Sample-14	C14O, C14P	Fatty Acid Synthase (FASN)
Sample-15	C21P	Fatty Acid Synthase (FASN)

*The location of each protein on the 2-D gel was defined by two parameters: the serum group and the location on the 384-plate. 1. The serum group is represented by the first letter, N represents Normal Serum, and C represents Cancer Serum. For example, N19A represents sample from Normal Serum and the PEP plate well 19A

### Biological verification with selected protein

**Fig. 7:**
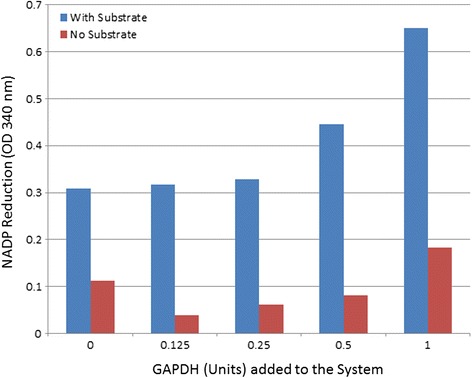
Purified GAPDH Increased Hexokinase Activity. To verify the proteins identified by mass spectrometry were indeed enhance the hexokinase activity, purified GAPDH was obtained from commercial source and added to the hexokinase activity assay system, and the enzyme activity was measured by measuring the NADP reduction at 340 nm

It has been reported that many genes from the glycolytic pathway are up-regulated [30, 39, 48] and reprogrammed metabolism is considered as a new hallmark of cancer development [19]. More interestingly, in addition to their role in mediating tumor growth through metabolism reprogramming, some of the metabolites have been shown to impact the signal transduction pathway [31], influence the tumor microenvironment or serving as a metabolic checkpoint for T cell response [8, 9, 21], all these recent findings suggest that the glycolytic pathway plays multiple roles in cancer development. As an example to demonstrate the proteins identified by MS is indeed enhancing the hexokinase activity, purified GAPDH from a commercial source was spiked into the hexokinase activity assay system. As can been seen in Fig. 7, the addition of purified GAPDH enhanced the hexokinase activity of the system, confirming the findings from the PEP plate and mass spectrometry analysis. Figure 7 also showed that the hexokinase activity enhancement is both GAPDH and hexokinase substrate-dependent, suggesting that the assay is mainly measuring the hexokinase product outcome instead of other enzymes that indirectly reduce NADP from the system. It will be interesting to further validate the other proteins identified from the system for their ability to enhance the hexokinase activity.

## Conclusions

Great effort has been made for the discovery and development of biomarkers for cancer diagnosis [11, 20, 24, 32, 40], however so far only a handful protein-based products have been approved with limited value especially in early cancer detection. One of the challenges in biomarker discovery has been the lack of disease-indicating biological features that can show high correlation with disease or disease stages. In cancer studies, it has long been recognized that cancer has significantly different metabolic behavior as compared with normal cells. Otto Warberg was the first to report the increased metabolic glucose activity in cancer tissue, there has been many reports linking increased metabolic activities with cancer development [27, 28, 33, 34, 38, 44, 49]. Recently there has been a surging interest in the development of drugs targeting enzymes from the glycolytic pathway [16–19, 21, 29, 39]. However, it is not clear how the whole glycolytic pathway and each of the components in the pathway contribute to the observed increase in glucose consumption. Using the PEP technology and the upstream enzyme, hexokinase, it is now possible to monitor the functions of many components in the glycolytic pathway and determine which components differentiate in the increased metabolic activity.

Human serum is very rich in proteins, thousands of proteins have been identified from human serum and many of them play important roles in maintaining homeostasis [7]. However so far no technology is available to systematically study the function of serum proteins, on the other hand, there is a possibility that the functions of these serum proteins could be strong indicators in many diseases. With the PEP technology, it is now possible to analyze the whole enzyme family or multiple enzyme families simultaneously so that a holistic understanding of the functional proteome of serum is possible. Using a pool of normal people serum and lung cancer patient serum, this study demonstrated that a large number of fractions with hexokinase activity could be detected, and the hexokinase activity was substrate and time-dependent. More interestingly, there were many fractions that showed significant activity difference between the normal and lung cancer patient, some of the activities are completely missing from the normal serum or vice versa. This is important for biomarker discovery and validation because human population could have very large individual variation in their composition of the serum [7].

In the current study, the enzyme assay system was designed to detect any rate-limiting enzyme in the glycolytic pathway because the first enzyme activity in the pathway, hexokinase, was selected. Therefore, any glycolytic enzymes downstream of hexokinase could enhance hexokinase kinetics by removing downstream products from the system. This could explain why GAPDH and other enzymes were identified from the highly active PEP wells. There are two important points need to be made from the current study: 1. that many time-dependent hexokinase activity fractions were detected from both the normal and lung cancer sera. This suggests that in human serum, there are many proteins that directly or indirectly influence hexokinase activity as measured by this assay. This also reflects the complexity of hexokinase regulation providing a potential panel of good biomarkers for diagnosis. 2. There are many hexokinase fractions that showed more than a 10-fold quantitative difference between the normal serum and lung cancer serum. This will make the development of baseline diagnostic monitoring easier because the detection has wide dynamic response, necessary for the large variation among the human population.

It is a little surprising that no gene products of hexokinase were identified from the fractions submitted for MS analysis. One possible reason is that hexokinase levels especially the active hexokinase levels in serum are very low or do not exist, since these activities are not normally extracellular. Therefore the observed hexokinase activity increase above basal level is likely to be an enhancement of the endogenous hexokinase activity from the beef liver extract, which itself contains a background level of hexokinase activity (shown in Fig. 7). In any case, the time-dependent and substrate-dependent increase in NADP reduction (the reporting signal) is at least an indication of activity enhancement by the protein(s) isolated from the PEP plate. Another possibility from the current finding is that the proteins identified have an alternative function other than their gene-designated functions. This can be further investigated in more detail with purified assay components and substrate.

Finally, we solicit that functional proteomics offers important insights to complex systems that otherwise could not be obtained. It is known from previous studies that human serum contains thousands of proteins [3] and it is speculated that many of them were leaked from human tissue, and thus may provide important clues to the health and status of the human body. Previous studies have shown that hexokinase activity is associated with cancer development, and recently an array of reports point to the importance of metabolic pathways in cancer development [4, 6, 10, 26]. In the current study, a novel technology - PEP, was used for the systematic analysis of glycolytic enzymes from human serum. Likewise, it is interesting to consider all metabolic enzymes as those that can be monitored and evaluated as possible biomarkers for staging overall well-being and disease. Even though only hexokinase activity analysis was reported here, enzyme assays on other families such as proteases, protein kinases and alkaline phosphatases were also performed on serum and major differences were also detected between normal and lung cancer patient serum (data not shown). So with these new methods to enrich and resolve functional proteins into multi-wells that can be profiled molecularly and compared, discoveries of important new lung cancer biomarkers are anticipated. Furthermore these same technologies can easily be extended for the development of biomarkers, drug targets or diagnostic kits for other types of cancer or disease.
